# Effect of Forest Therapy for Menopausal Women with Insomnia

**DOI:** 10.3390/ijerph17186548

**Published:** 2020-09-09

**Authors:** Hyeyun Kim, Jayoung Kim, Hyo Jin Ju, Bong Jin Jang, Tae Kyu Wang, Yeong In Kim

**Affiliations:** 1 Department of Neurology, Catholic Kwandong University, International St. Mary’s Hospital, Incheon 1600-8291, Korea; imkhy77@gmail.com; 2 Department of Laboratory Medicine, Catholic Kwandong University, International St. Mary’s Hospital, Incheon 1600-8291, Korea; lmkjy7@gmail.com; 3 College of Medicine, Catholic Kwandong University, International St. Mary’s Hospital, Incheon 1600-8291, Korea; 4 Graduate School of Healthcare Convergence, Catholic Kwandong University, International St. Mary’s Hospital, Incheon 1600-8291, Korea; jangbj@dhu.ac.kr; 5 Department of Public Administration, Catholic Kwandong University, Incheon 1600-8291, Korea; tkwang@changwon.ac.kr

**Keywords:** forest therapy, insomnia, sleep, menopause

## Abstract

Female hormone changes during menopause can affect the autonomic nervous system, circadian rhythm, and secretion of cortisol/melatonin, resulting in a vulnerability to insomnia. In this light, therapy has been gaining attention as a way to reduce stress hormones by stabilizing the autonomic nervous system. Thus, this study aims to objectively and scientifically analyze the impact of forest therapy in postmenopausal insomnia patients. The forest therapy program lasted 6 days, wherein 35 postmenopausal women performed activities such as trekking, leg massages, stretches, and bathing in warm and cold water. They also underwent serologic tests, participated in polysomnography (PSG), and answered sleep questionnaires before and after the program. Further, a statistical analysis compared the results. Serologic tests showed a significant reduction of cortisol from 10.2 ± 3.79 to 7.75 ± 2.81, while PSGs showed how sleep efficiency increased to 89.3 ± 4.3% (*p* < 0.01), and how waking after sleep onset reduced to 47.4 ± 22.3 min *(p <* 0.01). The total sleep time also increased to 428.5 min and sleep latency was 11.1 ± 11.0 min. Despite its limitations, forest therapy could be a good alternative to nonpharmacological treatment for mitigating insomnia in postmenopausal women.

## 1. Introduction

Despite numerous studies considering forest therapy as a form of alternative medicine, there remains insufficient scientific evidence because of varying forest compositions and healing or treatment programs. Therefore, various types of research on the effects of forest therapy were conducted to establish this form of therapy as an evidence-based alternative. One study verified the increased positive emotions of middle-aged women caused by forest therapy programs, reduced their negative emotions, such as depression and anxiety, and decreased their levels of serum cortisol after forest therapy [1]. In a study by Lee et al. [2], forest walking also showed increased cardiovascular relaxation and reduced negative emotional symptoms due to increased parasympathetic activity for young, healthy adult men. Forest therapy also showed positive effects on older adults diagnosed with hypertension, which lowered their blood pressure and cortisol concentration [3].

Although there are numerous studies on the effects of forest therapy, they mainly reported on positive emotional reinforcement and physical and psychological relaxation, with limited scientific bases. A previous study investigated the positive influence of forest therapy on sleep quality in 11 patients diagnosed with cancer, and it objectively and scientifically confirmed improved sleep efficiency and decreased sleep onset in the patients through polysomnography (PSG) [4]. 

Emotional fluctuation, psychological anxiety, and the aggravation of insomnia are caused by numerous factors that influence and interact with each other, such as hormones. In particular, menopause is a period when women experience hormonal, physiological, and emotional changes. In a survey of middle-aged women, up to 56% of the respondents reported sleep difficulties and insomnia [5]. Previous studies also report that hot flashes, low estrogen levels, and high follicle-stimulating hormones can cause insomnia [6,7]. 

This study intends to clarify the effects of forest therapy in the treatment of insomnia and to present its mechanism in menopausal women with relatively homogenous pathophysiologies. This study aimed to objectively and scientifically analyze the effectiveness of forest therapy programs in postmenopausal insomnia patients with similar physiologies. 

## 2. Materials and Methods 

### 2.1. Subjects

This study is a prospective, single-arm, single center clinical trial to evaluate the efficacy of forest therapy in postmenopausal women with insomnia. The study was conducted among women over the age of 40, who complained of insomnia caused by menopause. Menopause was diagnosed through an interview without any other clinical diagnosis by checking the regular menstruation. Insomnia was diagnosed by a clinician with the international classification of sleep disorder third edition.

Out of the 35 clinical participants, 25 were housewives, 6 were irregularly assigned to part-time jobs, and 4 volunteered at a church two or three times a week. Participants were physically active and capable of moderately intense exercise due to participate several programs during forest therapy. The volunteers who had depressive disorder, anxiety disorder, and psychiatric and cardiopulmonary disorders were excluded. Volunteers were screened through a questionnaire, a physical examination, and a blood test. 

### 2.2. The Design of Forest Therapy 

**Figure 1:**
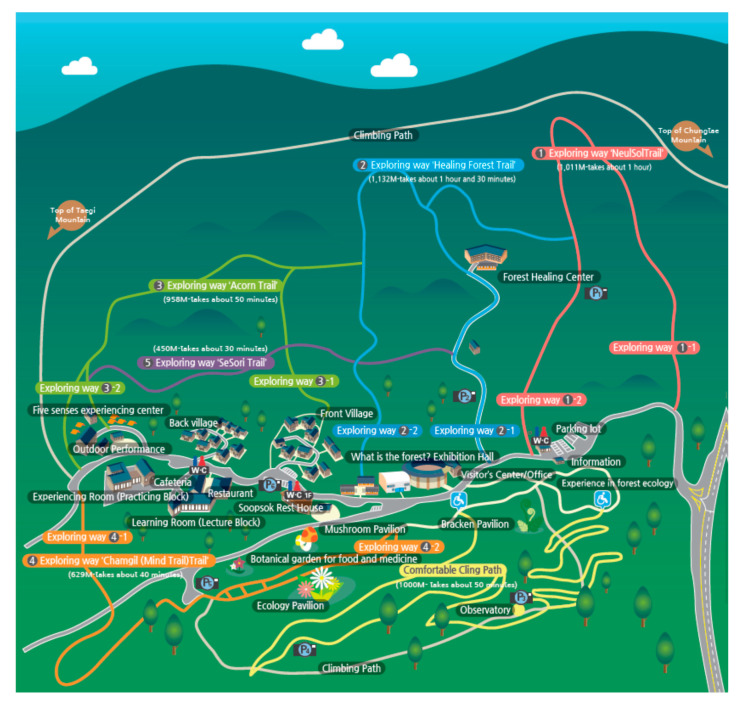
National center for forest activities in Hoengseong with trail routes.

The overall study lasted from June to October 2019 and had 35 participants; each program session lasted 6 days and had up to 6 participants. This program was conducted at the National Center for Forest Activities in Hoengseong, a forest education and healing center located 680 m above sea level (Figure 1). This location features 6 trails ranging from 450 to 2000 m in length, and trees such as larch, birch, dogwood, birch, and pine trees naturally grow here.

**Figure 2:**
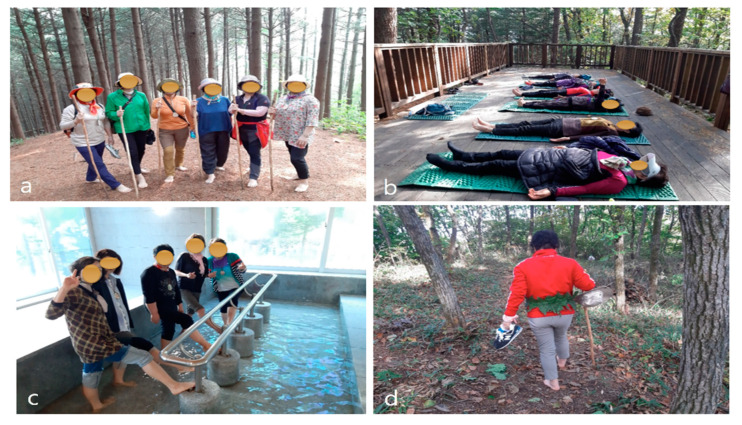
Participants performing various activities such as (**a**) barefoot trekking, (**b**) lying down in the forest, (**c**) foot massage with cold groundwater, and (**d**) feeling with the five senses.

Participants performed various activities, including meditation, gymnastics, 30 min morning forest walks before breakfast, and 1–2 h afternoon trail walks after lunch. The forest walks were performed at the same time, but the execution time showed a 20–30 min difference depending on each group’s activities and the weather. After the trail walk, participants were given leg massages and stretches, and alternately bathed in warm and cold water. The activity (or the Five Senses Experience Program) incorporates a wooden climbing stick woven with leaves around the waist and barefoot walks to maximize the use of the five senses (Figure 2). Participants were free to spend their free time in the forest and there were no restrictions other than leaving the forest. On the last night of the forest therapy program, PSG was conducted, and the cortisol test was performed at 8 a.m. before departure.

### 2.3. Evaluation of Sleep and Emotional Status, Laboratory Measurements 

Each participant’s sleep and emotional status was assessed using the Epworth sleepiness scale (ESS), the Stanford sleepiness scale (SSS) (to evaluate the daytime somnolence), the Korean version of the Pittsburgh Sleep Questionnaire Index (PSQI) for sleep quality investigation, insomnia severity index for measure the severity of insomnia symptoms, the STOP-Bang (to evaluate sleep apnea), the Hospital Anxiety and Depression Scale (HADS), and PSG before and after the forest therapy program [8,9,10,11,12]. Blood samples obtained before and after forest therapy for general evaluation examined various elements, including common blood cells (CBCs), glucose, aspartate transaminase (AST), alanine transaminase (ALT), blood urine nitrogen, creatinine, electrolytes such as sodium, potassium, chloride, C-reactive protein (CRP), interleukin 6, and cortisol. In the case of cortisol, blood was sampled at 8 a.m. because it has circadian changes.

PSG was conducted before and after forest therapy, but baseline PSG was conducted one week before participation in the sleep lab of the research institute. PSG was performed using a digital PSG machine (Nox A1, Nox Medical Inc., Reykjavik, Iceland). The following variables were monitored: electroencephalogram (EEG; C3-A2, C4-A1, O2-A1, O1-A2), right and left electro-oculogram, submental, both anterior tibialis electromyograms, electrocardiogram, airflow (pressure cannula and thermistor), respiratory effort (piezo-electric bands), oxyhemoglobin saturation (SaO_2_), and snoring [13].

This study was conducted following the Declaration of Helsinki and the protocols were approved by the appropriate ethics review board (#IS19EISE0024). All participants gave their consent before participating in the study.

### 2.4. Statistical Analysis

All values from this study are presented as the mean ± standard variations. In the case of this study, the paired *t*-test was used because the normal distribution was shown as a result of the normality test of the sample. The normality test was confirmed through the test results of Kolmogorov–Smirnov. The difference between the mean values of the variables including the results of serology, questionnaire and PSG before and after forest therapy was analyzed using the paired *t*-test. A *p* value of less than 0.05 was considered statistically significant. All analyses were conducted using SPSS software (version 13.0 SPSS, Inc., Chicago, IL, USA).

## 3. Results

The average age of all the participants was 58.8 ± 3.9 (mean ± standard deviation) years old and the physical examination and baseline serologic test results are presented in Table 1. Table 2 showed the immunologic and stress hormone results before and after forest therapy, while cortisol levels showed a statistically significant reduction from 10.2 ± 3.79 mcg/dL to 7.75 ± 2.81 mcg/dL. There were no significant changes in interleukin 6 and C-reactive protein (CRP) levels.

The results of the sleep questionnaire are as follows (Table 3). The ESS, which evaluates daytime sleepiness, improved from 7.4 ± 4.7 to 6.0 ± 3.8, and the anxiety and depression index improved from 6.7 ± 3.3 to 5.5 ± 3.2, and from 5.4 ± 2.5 to 4.5 ± 2.5, respectively. ISI showed a numerical improvement from 12 ± 5.2 to 11 ± 5.5, but this did not show statistical significance. The ESS, which evaluates sleepiness in a specific situation, is judged as out of normal sleepiness with a score of 11 or higher. The number of patients who complained of daytime sleepiness through ESS evaluation decreased from 8 to 4 before forest therapy. In addition, 4 out of 5 participants who reported having nighttime hot flashes reported no hot flashes during the forest therapy.

In the PSG results, sleep efficiency increased from 76.9 ± 4.8% to 89.3 ± 4.3% after forest therapy *(p <* 0.01). Because sleep efficiency of at least 85% is considered normal, the representative symptom of insomnia improved with forest therapy. Waking after sleep onset also showed a reduction from 95.5 ± 42.1 min to 47.4 ± 22.3 min *(p <* 0.01). Total sleep time was not statistically significant but increased from 409.7 min to 428.5 min and sleep latency was reduced to 11.1 ± 11.0 min (Table 4).

## 4. Discussion

Insomnia is a highly prevalent sleep disorder and treatment methods could be divided into pharmacological (sleeping pills and herbal or nutritional supplements) and nonpharmacological therapies (acupuncture, high-intensity exercise, and yoga). Here, nonpharmacological treatment of insomnia should be considered first when considering addictions and other side effects because some proposed methods are similar to drug treatment. This study, however, proposes the use of forest therapy as a nonpharmacological treatment method.

Forest therapy is an actively researched topic of alternative medicine mainly used in Japan, but its scientific basis is insufficient despite yielding positive results. Recently, studies about forest therapy have been actively conducted from an evidence-based perspective. Walks in the forest improved cardiovascular relaxation and reduced negative emotional symptoms in healthy, young adult males due to an increase in parasympathetic nerve activity [2]. A study conducted on elderly, hypertensive patients objectively verified the effect of forest therapy in improving the quality of life by reducing blood pressure and cortisol levels [3]. A forest therapy program for middle-aged women also reported an increase in positive emotions and a reduction in cortisol concentrations [1]. It is known to be a change accompanied by an elevated cortisol in premenopausal and postmenopausal, which shows rapid hormonal changes. It is reported that it is also related to loss of circadian robustness. Ref. [14] Loss of circadian robustness directly affects sleep quality. In summary, it has a vicious cycle that leads to menopause, increased cortisol secretion, loss of circadian rhythm, and insomnia. Forest therapy for insomnia, which can induce a decrease in the secretion of cortisol, might be one of the ways to break this vicious cycle. In 2018, the effectiveness of a forest therapy program for patients diagnosed with cancer was verified through a preliminary study, which showed increased sleep efficiency and sleep initiation times through polysomnographic findings [4]. After reviewing previous studies about forest therapy and nonpharmacological treatments for insomnia and menopause, various activities for relaxing and meditating in the forest are expected to have synergistic effects in insomnia and menopause treatment. As a result, forest therapy increased sleep efficiency and reduced the frequency of interrupted sleep.

A cross-sectional observational study of 339 perimenopausal women with high levels of recreational or sports physical activity was associated with significantly lower complaints of insomnia. A prospective within-group pilot study of 12 women who engaged in 10 weekly Hatha yoga sessions reported modest reductions in hot flashes and insomnia. In an open-label study of seven women with menopausal symptoms and chronic insomnia, a one-hour therapeutic massage conducted twice a week improved the subjects’ sleep quality. There have been a few nonpharmacological treatment studies in menopausal women with insomnia, but the previous studies showed reductions in hot flashes and insomnia. This study can verify the positive effect similar to the previous study, showing how forest therapy for postmenopausal women with insomnia could be a nonpharmacological treatment option.

However, to our knowledge, there is no study detailing how long staying in a forest is effective or not. Through the experience of our prior research, participants were unable to sleep on the first day of the program due to unfamiliar sleeping environments [4]. Based on several previous trials of forest therapy, our program’s duration was set at five nights and six days [1,2,3,15]. Moreover, this study prioritized proving forest therapy’s efficacy for insomnia to develop an efficient and scientifically backed protocol, especially for controlling the sleeping environments of insomniac participants. Therefore, the duration participants stayed in the forest was an essential consideration for developing forest therapy because the symptoms of insomnia were exacerbated in the new sleep environment. Based on the study’s results, forest therapy programs should last five nights and six days for postmenopausal women with insomnia. However, more studies are needed to establish standardized therapies because the influence of sleep environments on insomniacs varies from patient to patient.

While planning the study, activities that double the therapy’s effectiveness were considered and the activities were chosen based on several criteria. First, the activities (meditating, walking barefoot, etc.) should stimulate the five senses because the various effects of the therapy must result in the synergistic effect of the forest’s positive energy instead of only one effect [16]. Second, forest therapy activities should utilize healthy light. In illuminated, high-altitude forests, exposure to healthy sunlight during the day could have a positive effect on nighttime sleep; activities chosen included those with exposure to healthy light as well as those performed in the shade. Third, activities should consider the physical strength of each participant.

The study recruited participants of similar age, gender, and physiology, which made activities more accessible. For example, participants who could not walk long distances were guided along shorter trails. Lastly, the chosen activities should focus on relaxing the participant’s mind and body while alleviating insomnia. Among the main activities selected for this purpose are meditation, gymnastics, and water therapy.

This study, however, had several limitations. First, verifying the effect of the forest on the study participants was difficult. Although previous participant interviews determined the activities suitable for forest therapy, this process was ineffective; studies comparing the effects of activities performed in the city and the forest are necessary. Second, environmental factors such as temperature, humidity, and illuminance, which are required for good sleep hygiene, were not controlled. However, it was impossible to rule out the possibility that a controlled environment contributed to the alleviation of insomnia. Third, educating participants on how they can sleep better before participating in the study may have affected the study. All participants were educated on the topic a week before undergoing forest therapy because the study aimed to improve sleep quality through better sleep hygiene and behavior with a firm understanding of the relationship between menopause and sleep. Fourth, the hormonal effects of postmenopausal women were not standardized in this study. The study participants were women who complained of insomnia that occurred or worsened after menopause. Subjects experiencing hot flashes were selected for the study, but their estrogen and progesterone levels were not measured. Lastly, the study was conducted without a control group. A control group will be needed for clarification. Moreover, the primary treatment for chronic insomnia is cognitive behavioral therapy. Among the known cognitive behavioral treatments, exposure to sunlight during the day for sleep hygiene, proper exercise, and regular diet were applied during this study. Therefore, forest therapy could not be suggested as an alternative to cognitive behavioral therapy. Despite these limitations, this may be the first study that attempted to verify the effectiveness of forest therapy for insomnia using PSG.

## 5. Conclusions

Through this prospective clinical study, it can be determined that forest therapy may be an effective alternative to nonpharmacological treatment for insomnia, especially in postmenopausal women.

## Figures and Tables

**Table 1:** General characteristics of the study participants.

Variables	Mean ± Standard Deviation
Age (years old)	58.8 ± 3.9
Height (cm)	155.8 ± 4.2
Weight (Kg)	58.32 ± 8.8
BMI(Kg/m^2^) ^1^	23.4 ± 5.0
AST (IU/L) ^2^	24.7 ± 5.5
ALT (IU/L) ^3^	23.6 ± 8.0
BUN (mg/dL) ^4^	15.7 ± 3.8
Creatine (mg/dL)	0.6 ± 0.1
Na (mmol/L)	139 ± 1.7
K (mmol/L)	5.2 ± 6.2
Cl (mmol/L)	106.3 ± 1.6
Glucose (mg/dL)	102.8 ± 11.3
CRP (mg/L) ^5^	1.0 ± 1.6
Hemoglobin (g/dL)	13.0 ± 1.2

^1^ Body Mass Index, ^2^ Apartate transaminase, ^3^ Anine transaminase, ^4^ Blood urine nitrogen, ^5^ C-reactive protein.

**Table 2:** Immunologic and stress hormone levels before and after forest therapy.

Hormones	Before Forest Therapy	After Forest Therapy	*p*
Interleukin 6 (pg/mL)	3.16 ± 1.11	2.80 ± 0.89	0.628
CRP (mg/L)	1.08 ± 1.63	0.98 ± 0.94	0.597
Cortisol (mcg/dL)	10.2 ± 3.79	7.75 ± 2.81	0.005

**Table 3:** Questionnaire results before and after forest therapy.

Variables	Before Forest Therapy	After Forest Therapy	*p*
PSQI-K ^1^	9.7 ± 3.3	9.1 ± 3.5	0.298
STOP-Bang	2.4 ± 1.2	2.7 ± 1.2	0.298
SSS ^2^	2.8 ± 1.2	2.6 ± 1.1	0.490
ESS ^3^	7.4 ± 4.7	6.0 ± 3.8	0.207
HADS ^4^ Anxiety	6.7 ± 3.3	5.5 ± 3.2	0.457
HADS Depression	5.4 ± 2.5	4.5 ± 2.5	0.157
ISI ^5^	12 ± 5.2	11 ± 5.5	0.444

^1^ Pittsburg Sleep Quality Scale-K, ^2^ Stanford Sleepiness Scale, ^3^ Epworth Sleepiness Scale, ^4^ Hospital Anxiety and Depression Scale, ^5^ Insomnia Severity Index.

**Table 4:** Polysomnography findings before and after forest therapy.

Variables	Before Forest Therapy (N = 35)	After Forest Therapy (N = 35)	*p*
Total sleep time (min)	409.7 ± 51.2	428.5 ± 57.3	0.207
Latency to sleep onset (min)	25.3 ± 26.6	11.1 ± 11.0	0.063
Latency to REM ^1^ onset (min)	108.9 ± 82.7	92.7 ± 63.2	0.537
Sleep efficiency (%)	76.9 ± 4.8	89.3 ± 4.3	<0.01
Sleep stage N1 (%)	4.5 ± 4.5	5.2 ± 5.0	0.869
Sleep stage N2 (%)	64.1 ± 32.2	67.4 ± 55.0	0.139
Sleep stage N3 (%)	18.1 ± 11.0	21.4 ± 12.5	0.157
REM (%)	21.6 ± 12.3	28.6 ± 26.3	0.160
Waking after sleep onset (min)	95.5 ± 42.1	47.4 ± 22.3	<0.01
AHI ^2^ (/h)	20.3 ± 14.5	23.9 ± 13.6	0.104
PLMI ^3^ (/h)	21.6 ± 31.4	21.1 ± 40.6	0.698
ODI ^4^ (/h)	13.9 ± 15.7	18.1 ± 12.8	0.007

^1^ Rapid eye movement, ^2^ Apnea Hypopnea Index, ^3^ Periodic Limb Movements Index, ^4^ Oxygen desaturation index.
